# Supplementary material for: Effects of Rumen-Protected β-Alanine on Growth Performance, Rumen Microbiome, and Serum Metabolome of Beef Cattle
Source: Animals (Basel). 2025 Dec 24;16(1):43. doi: 10.3390/ani16010043 (PMC12785137; doi:10.3390/ani16010043)
Supplement: Supplementary file 1 [file animals-16-00043-s001.zip › Table S1 Summary of sequenced sequences of rumen fluid from beef cattle.pdf]

Table S1 Summary of sequenced sequences of rumen fluid from beef cattle

Samples	Raw reads	Sequences
A1	761746	501768
A2	638837	352350
A3	766013	462150
A4	790913	487791
A5	857518	462286
A6	714218	408347
B1	691044	416357
B2	584831	364719
B3	698252	395765
B4	721726	470739
B5	907855	620675
B6	661500	377349
Total	8794453	5320296
Mean	732871	443358
SD	90819	74670
SEM	26217	21555
