# Supplementary figures and images for: Effects of Rumen-Protected β-Alanine on Growth Performance, Rumen Microbiome, and Serum Metabolome of Beef Cattle
Source: Animals (Basel). 2025 Dec 24;16(1):43. doi: 10.3390/ani16010043 (PMC12785137; doi:10.3390/ani16010043)

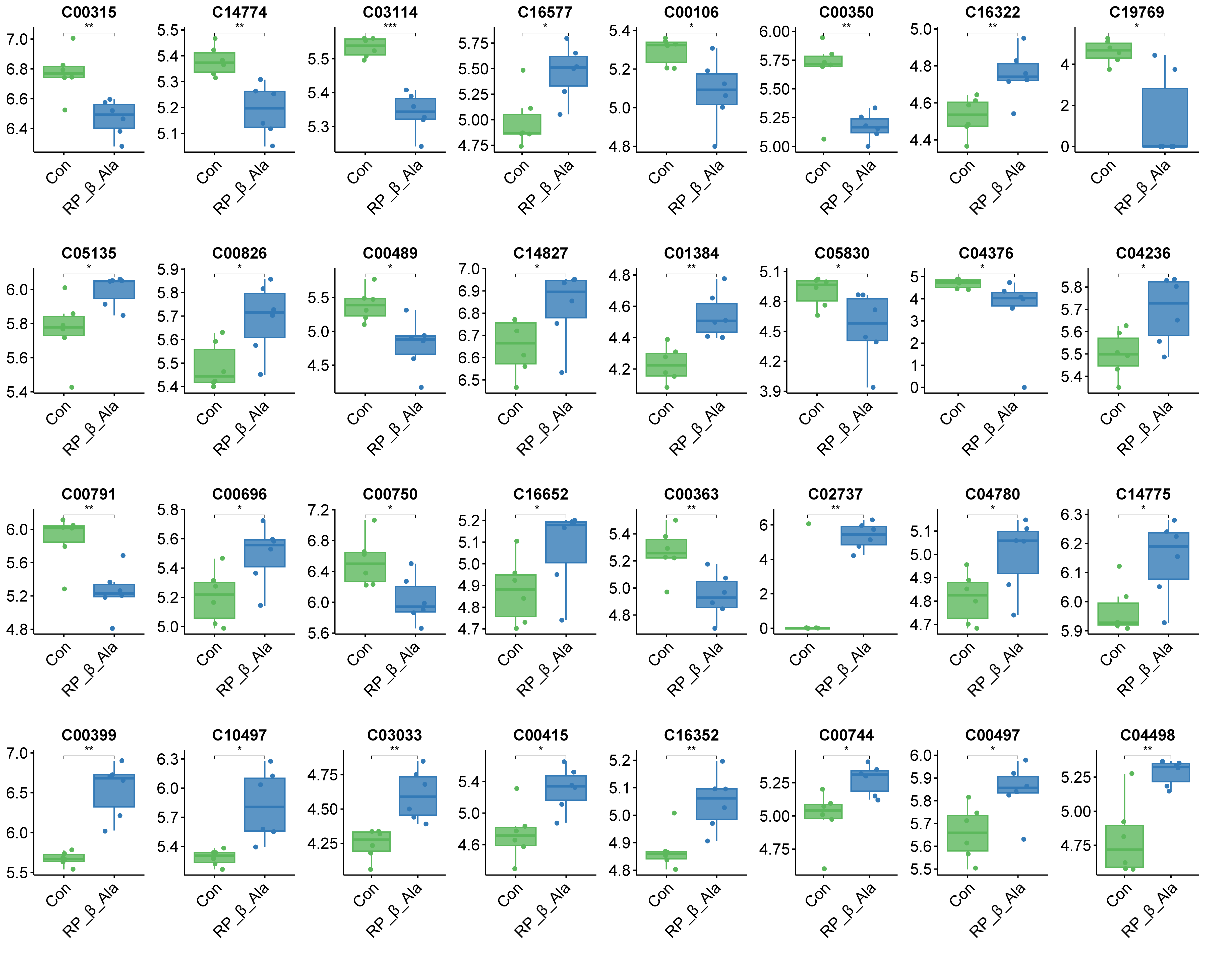

Supplement: Supplementary file 1 [file animals-16-00043-s001.zip › Fig. S1 Violin plot analysis of 32 differential metabolites between control and RP-β-Ala groups.png]
